# A Co-Association Network Analysis Reveals Putative Regulators for Health-Related Traits in Pigs

**DOI:** 10.3389/fimmu.2021.784978

**Published:** 2021-11-26

**Authors:** Daniel Crespo-Piazuelo, Yuliaxis Ramayo-Caldas, Olga González-Rodríguez, Mariam Pascual, Raquel Quintanilla, Maria Ballester

**Affiliations:** Animal Breeding and Genetics Programme, Institut de Recerca i Tecnologia Agroalimentàries (IRTA), Torre Marimon, Caldes de Montbui, Spain

**Keywords:** immunocompetence, pig, gene networks, transcription factors, systems biology, candidate genes

## Abstract

In recent years, the increase in awareness of antimicrobial resistance together with the societal demand of healthier meat products have driven attention to health-related traits in livestock production. Previous studies have reported medium to high heritabilities for these traits and described genomic regions associated with them. Despite its genetic component, health- and immunity-related traits are complex and its study by association analysis with genomic markers may be missing some information. To analyse multiple phenotypes and gene-by-gene interactions, systems biology approaches, such as the association weight matrix (AWM), allows combining genome wide association study results with network inference algorithms. The present study aimed to identify gene networks, key regulators and candidate genes associated to immunocompetence in pigs by integrating multiple health-related traits, enriched for innate immune phenotypes, using the AWM approach. The co-association network analysis unveiled a network comprised of 3,636 nodes (genes) and 451,407 edges (interactions), including a total of 246 regulators. From these, five genes (*ARNT2*, *BRMS1L*, *MED12L*, *SUPT3H* and *TRIM25*) were selected as key regulators as they were associated with the maximum number of genes with the minimum overlapping (1,827 genes in total). The five regulators were involved in pathways related to immunity such as lymphocyte differentiation and activation, platelet activation and degranulation, megakaryocyte differentiation, FcγR-mediated phagocytosis and response to nitric oxide, among others, but also in immunometabolism. Furthermore, we identified genes co-associated with the key regulators previously reported as candidate genes (e.g., *ANGPT1, CD4, CD36, DOCK1*, *PDE4B, PRKCE, PTPRC* and *SH2B3*) for immunity traits in humans and pigs, but also new candidate ones (e.g., *ACSL3*, *CXADR*, *HBB*, *MMP12*, *PTPN6*, *WLS*) that were not previously described. The co-association analysis revealed new regulators associated with health-related traits in pigs. This approach also identified gene-by-gene interactions and candidate genes involved in pathways related to cell fate and metabolic and immune functions. Our results shed new light in the regulatory mechanisms involved in pig immunity and reinforce the use of the pig as biomedical model.

## Introduction

Health-related traits are becoming more and more relevant due to the great challenges that the pig sector has faced in recent years. The current emergence of antibiotic resistance, with pig production as one of the largest consumers of antimicrobials, and the societal demand for healthier livestock products and more sustainable production systems, make necessary to include new traits such as those related to health in the selection programs (1). Thus, health-related traits that have traditionally played a minor role in breeding programs are currently considered relevant traits to take into account.

Several studies have determined an important genetic contribution to the phenotypic variability of health-related traits, describing medium to high heritabilities for some of these traits (2–7) and identifying genomic regions with candidate genes associated to immunocompetence (7, 8). However, although genome wide association studies (GWAS) have identified single nucleotide polymorphisms (SNPs), or genes, associated with health-related traits, these SNPs generally had a little effect on the phenotypic variation of the traits under study due to their complex nature. Besides, some interesting SNPs could be overlooked due to stringent significance thresholds after multiple test correction (9). Furthermore, these studies do not consider gene-by-gene interactions or the contribution of genetic correlations between related traits.

During the last years, the use of systems biology approaches has allowed the integration of multiple sources of biological information, providing a more complete picture of the functional mechanisms involved in the determination of complex traits, and allowing the identification of candidate genes and genetic variants implicated in their phenotypic diversity (10). One of these approaches, the association weight matrix (AWM), simultaneously considers multiple phenotypes and gene-by-gene interactions by combining GWAS results with network inference algorithms (11, 12). To date, multiple studies in livestock species have implemented this approach to identify gene networks and key regulatory elements implicated in the genetic architecture of meat quality (13, 14), growth (15), gut microbial communities (16), reproductive (11, 17), feed efficiency (18, 19), and milk-related (20–23) traits, among others. However, to the best of our knowledge, no studies have applied this methodology for health-related (mainly immunological and haematological) traits in livestock or other animal species.

The present study aimed to identify gene networks, key regulators and candidate genes associated to immuno-competence in pigs by integrating 30 health-related traits, enriched for innate immune phenotypes, using the AWM co-association analysis.

## Material and Methods

### Animal Material and Phenotypic Parameters

The animal material used in this study was previously reported in Ballester et al. (7). In brief, we used a total of 432 weaned piglets (217 males and 215 females) from a commercial Duroc pig line. The pigs were distributed in six batches and belonged to 134 litters obtained from 132 sows and 22 boars. All animals were raised in the same farm and fed *ad libitum* with a commercial cereal-based diet. At the time of sample collection (60 ± 8 days of age), all animals were apparently healthy, without any sign of infection.

Details of sampling and laboratory determination of immunological, haematological and stress parameters are described in Ballester et al. (7). In brief, total concentrations of immunoglobulins IgA, IgG and IgM in plasma, and IgA in saliva (IgAsal), were measured by ELISA with commercial kits (Bethyl laboratories Inc., Bionova, Spain), following manufacturer’s instructions. Concentration of acute phase proteins: C-reactive protein (CRP) and haptoglobin (HP) were measured in serum by ELISA kit (Abcam Plc., Spain) and colorimetric assay (Tridelta Development Limited, Ireland), respectively, following manufacturer’s instructions. Total concentration of nitric oxide (NO) in serum was measured by colorimetric assay (Termo Fisher Scientifc, Spain). To determine the percentage of gamma-delta T cells (γδ T cells), peripheral blood mononuclear cells (PBMCs) were separated from heparinized peripheral blood by density-gradient centrifugation with Histopaque-1077 (Sigma, Spain) and 10^6^ PBMCs were stained using the monoclonal antibody APC Rat Anti-Pig γδ T Lymphocytes (MAC320 clone, BD Pharmigen, Spain) and the APC Rat IgG2a κ isotype control (R35-95 clone, BD Pharmigen, Spain). The cells were analysed by flow cytometry using the MACSQuant Analyzer 10 Flow cytometer (Miltenyi Biotec GmbH, Bergisch Gladbach, Germany) and the MACSQuantify sofware v2.6 (Miltenyi Biotec GmbH, Bergisch Gladbach, Germany). Phagocytosis assay was carried out in heparinized whole blood samples incubated with fluorescein (FITC)-labelled opsonized *E. coli* bacteria by using the Phagotest kit (BD Pharmigen, Spain) according to manufacturer’s instructions. The following percentages of phagocytosis traits in blood: percentage of total phagocytic cells (PHAGO_%); percentage of phagocytic cells among granulocytes (GRANU_PHAGO_%), monocytes (MON_PHAGO_%) and lymphocytes (LYM_PHAGO_%); mean fluorescence in fluorescein isothiocyanate (FITC) among the total phagocytic cells (PHAGO_FITC); and mean fluorescence in FITC among the granulocytes (GRANU_ PHAGO_FITC), monocytes (MON_PHAGO_FITC) and lymphocytes (LYM_PHAGO_FITC) that phagocyte were analysed by flow cytometry (MACSQuant Analyzer 10 Flow cytometer; Miltenyi Biotec GmbH, Bergisch Gladbach, Germany) and the MACSQuantify sofware v2.6 (Miltenyi Biotec GmbH, Bergisch Gladbach, Germany). Haematological traits: haematocrit (HCT), haemoglobin (HB), mean corpuscular volume (MCV), mean corpuscular haemoglobin (MCH), mean corpuscular haemoglobin concentration (MCHC), total number of leukocytes (LEU), eosinophils (EO), lymphocytes (LYM), monocytes (MON), neutrophils (NEU), erythrocytes (ERY) and platelets (PLA) were determined from blood samples by haemogram. Finally, the stress indicators analysed were the neutrophil to lymphocyte ratio (NLR) and the cortisol (CORT) levels in hair, which were measured by ELISA kit (Cusabio Technology LLC., Bionova, Spain). **Table S1** summarizes the descriptive statistics of the analysed traits.

### SNP Genotyping and Genome Wide Association Analysis

The 432 animals were genotyped with the GGP Porcine HD Array (Illumina, San Diego, CA) using the Infinium HD Assay Ultra protocol (Illumina). The quality control of the 68,516 SNPs was performed using Plink software (24), removing SNPs with a minor allele frequency lower than 5%, SNPs with more than 10% missing genotype data, and SNPs that did not map to the porcine reference genome (*Sscrofa11.1* assembly). Thereafter, a subset of 42,641 SNPs was retained for subsequent analysis.

GWAS for the 30 health-related traits was performed using the following mixed linear model with the Genome-wide Complex Trait Analysis (GCTA) software tool (25):


$$y_{ijk}=sex_{j}+b_{k}+u_{i}+s_{li}a_{l}+e_{ijk}$$

where *y_ijk_* corresponds to the phenotypic trait (either log-transformed or raw data) of the i^th^ individual of sex j in the k^th^ batch; *sex_j_* corresponds to the j^th^ sex effect (2 levels); *b_k_* corresponds to the k^th^ batch effect (6 levels) for most traits but for phagocytosis related traits, for which the data of laboratory analysis (12 levels, two by batch) was considered instead; *u_i_* is the infinitesimal genetic effect of individual i, with u∼N(0,G $\sigma_{u}^{2}$ ), where G is the genomic relationship matrix calculated using the filtered autosomal SNPs based on the methodology of (25) and $\sigma_{u}^{2}$ is the additive genetic variance; *s_li_* is the genotype (coded as 0,1,2) for the l^th^ SNP, and a_l_ is the allele substitution effect of the SNP on the trait under study; and *e_ijk_* is the residual term.

The GCTA software tool (25) was also used to estimate the heritability value for each one of the 30 phenotypes by performing a restricted maximum likelihood (REML) analysis with sex and batch as fixed effects.

### Association Weight Matrix

The results from GWAS analysis were subjected to an AWM approach (11, 12). For the initial step, only those SNPs located in the coding region or within 5 kb of an annotated gene based on the *Sscrofa11.1* reference genome assembly were kept. Then, γδ T cells was chosen as key phenotype and those γδ T cells-associated SNPs (*P*<0.05) were selected. In addition, we also retained those SNPs that were associated (*P*<0.05) with three or more of the 30 health-related phenotypes and referred to as pleiotropic SNPs. For assessing the gene-gene interactions, the standardized SNP effects across phenotypes were used to infer the gene co-association network using the Partial Correlation and Information Theory (PCIT) approaches (26). In the network, every node represents a gene (or a SNP), whereas every edge connecting two nodes represents a significant gene–gene interaction (based on SNP–SNP co-association). In order to quantify the number of connections as well as to prioritize potential regulators of the gene network we applied an information lossless approach (27) that explored the connectivity of all regulators (transcription factors, miRNAs and lnRNAs) and its target genes. The list of the regulators that was cross-matched with the list of 3,636 genes corresponds to the census of transcription factors reported by Vaquerizas et al. (28). The Cytoscape software (29) was used to visualize the gene network and calculate node centrality values.

### Functional and Comparative Analyses

When only the Ensembl ID was available for some of the associated novel genes, they were searched against the database for human orthologues (*GRCh38.p13*) to extract their putative gene name using the BioMart web-based tool (30).

ClueGO plugin (31) was used to identify the over-represented gene ontology terms, KEGG pathways, and immune functions. The cut-off for considering a significant over-representation (corrected *p*-value ≤ 0.05) was established by Benjamini and Hochberg multiple-test correction (32). Further categorization of candidate genes was performed using information from the Mouse Genome Database (www.informatics.jax.org) (33) and GeneCards (www.genecards.org) (34).

A comparative analysis between our results and previous published data was performed by retrieving all pig and human quantitative trait loci (QTLs) and association data on *Sscrofa11.1* and on *GRCh38.p13* (dbSNP Build 153), for health traits from the pigQTL database (35) and from the NHGRI-EBI GWAS Catalog (v1.0.2) (36), respectively.

## Results

### Gene Co-Association Network Description

From the 42,641 SNPs used in the GWAS analysis, 2,230 SNPs were directly associated (*P*<0.05) with the key phenotype (γδ T cells), while 8,421 SNPs were associated (*P*<0.05) with three or more phenotypes. Thus, a total of 9,830 SNPs were obtained after merging both lists of associated SNPs. Two SNPs located in *Sus scrofa* chromosome (SSC) 13 (*rs323669145* and *rs81448146*) were associated with the maximum number of phenotypes, 13 out of the 30 traits (CRP, HP, NO, CORT, MCHC, LYM, NLR, PHAGO_%, PHAGO_FITC, GRANU_PHAGO_%, GRANU_ PHAGO_FITC, MON_PHAGO_FITC, LYM_PHAGO_%). Those two SNPs were located in intronic regions of the fibroblast growth factor 12 (*FGF12*) gene. Among other SNPs associated with a high number of phenotypes (n≥10) we also identified *rs81284215* on SSC9. Remarkably, this SNP was located in the haemoglobin subunit beta (*HBB*) gene and was associated with haematological-related phenotypes such as HCT, HB and ERY, as well as with γδ T cells and six out of the eight phagocytosis phenotypes, excluding those related to lymphocytes.

After the annotation step, only 3,544 out of the 9,830 SNPs were retained as they were located in the coding region or 5 kb upstream or downstream of a gene. However, as some SNPs were annotated to more than one gene, a total of 3,650 unique genes were identified. To explore the genetic determinism of the 3,544 SNPs selected from the AWM approach over the 30 phenotypes, their heritability was calculated using these SNPs and compared to the heritabilities obtained using the whole dataset comprised by 42,641 SNPs (**Table 1**). Heritability calculated with the SNPs subset ranged between 0.238 and 0.791. The highest heritabilities (h^2^>0.7) were observed for the haematological traits (MCH, MCV, HB, HCT), while CRP, MON, and IgAsal showed low heritabilities (h^2^<0.3). However, the other immunoglobulin-related traits (IgG, IgA and IgM) had heritabilities above 0.5. In addition, most of the traits (24 out of 30) showed moderate to high heritabilities (h^2^>0.4). When compared with the heritabilities obtained with the whole dataset, the heritabilities of the SNPs subset showed higher values in all comparisons except one (LYM_PHAGO_FITC), whose values were almost similar (0.374 vs. 0.379). The greatest improvement in the heritability value was observed for HB and HCT, while others, such as the values for CRP, IgG, and the aforementioned LYM_PHAGO_FITC, did not change much. Of note, the heritability of the key phenotype (γδ T cells) was also greatly improved from moderate in the whole dataset (0.439) to high in the subset (0.688). Furthermore, to validate the heritabilities obtained by the reduced dataset of 3,544 SNPs, the heritability values of the 30 phenotypes were calculated by randomly selecting the same number of SNPs from the whole dataset in 100 different iterations. Thus, for each phenotype, the heritability values of the reduced dataset were always higher than the heritability values randomly obtained with each iteration with the exception, again, of the LYM_PHAGO_FITC phenotype.

**Table 1:** Heritability values (h^2^), and their standard errors (SE), for the immunological, haematological and stress analysed traits obtained using the 3,544 SNPs selected from the AWM approach and the whole dataset (42,641 SNPs).

Trait	3544 SNPs		Whole dataset	
	h^2^	SE	h^2^	SE
**Haematocrit (%)**	0.704	0.0631	0.338	0.0934
**Haemoglobin (g/dL)**	0.739	0.0603	0.371	0.0917
**Erythrocytes count (n/µL)**	0.688	0.0613	0.532	0.0796
**Mean corpuscular volume (fL)**	0.778	0.0517	0.588	0.0858
**Mean corpuscular haemoglobin (pg)**	0.791	0.0505	0.560	0.0887
**Mean corpuscular haemoglobin concentration (g/dL)**	0.635	0.0735	0.503	0.0983
**Platelets count (n/µL)**	0.531	0.0777	0.449	0.0927
**Leukocytes count (n/µL)**	0.518	0.0784	0.226	0.0801
**Eosinophils count (n/µL)**	0.505	0.0803	0.345	0.0875
**Lymphocytes count (n/µL)**	0.614	0.0733	0.314	0.0912
**Monocytes count (n/µL)**	0.279	0.0802	0.076	0.0585
**Neutrophils count (n/µL)**	0.503	0.0768	0.299	0.0853
**IgA in saliva (mg/dl)**	0.284	0.0869	0.188	0.0878
**IgA in plasma (mg/ml)**	0.607	0.0716	0.497	0.0867
**IgG in plasma (mg/ml)**	0.618	0.0734	0.593	0.0840
**IgM in plasma (mg/ml)**	0.542	0.0758	0.399	0.0817
**C-reactive protein in serum (µg/ml)**	0.238	0.0796	0.180	0.0797
**Haptoglobin in serum (mg/ml)**	0.441	0.0828	0.285	0.0890
**Nitric oxide in serum (µM)**	0.365	0.0831	0.278	0.0895
**γδ T-lymphocytes subpopulation (%)**	0.688	0.0666	0.439	0.0895
**Phagocytosis (% cells)**	0.488	0.0777	0.284	0.0852
**Granulocytes phagocytosis (%)**	0.455	0.0812	0.285	0.0897
**Monocytes phagocytosis (%)**	0.582	0.0768	0.368	0.0929
**Lymphocytes phagocytosis (%)**	0.622	0.0694	0.531	0.0845
**Phagocytosis FITC**	0.507	0.0741	0.406	0.0855
**Granulocytes phagocytosis FITC**	0.542	0.0747	0.416	0.0870
**Monocytes phagocytosis FITC**	0.435	0.0793	0.308	0.0913
**Lymphocytes phagocytosis FITC**	0.374	0.0855	0.379	0.0972
**Cortisol in hair (pg/mg)**	0.344	0.0842	0.209	0.0821
**NEU/LYM ratio**	0.619	0.0709	0.466	0.0945

**Figure 1:**
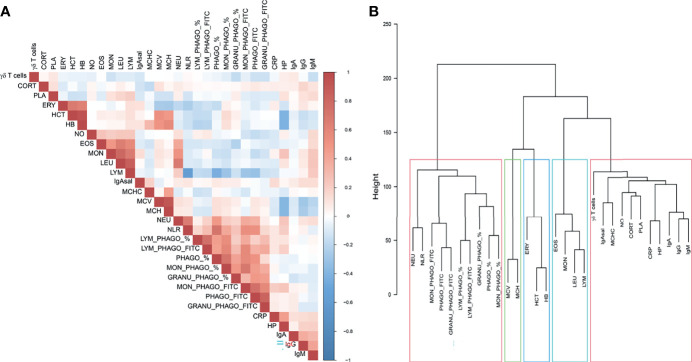
Genetic relationships among phenotypes based on the normalized additive values of the 3,544 SNPs selected for the AWM approach. **(A)** Heatmap of the correlations estimated by pairwise combinations and **(B)** Hierarchical cluster analysis among immunity, haematological and stress related traits in pigs.

Genetic relationships among phenotypes based on the normalized additive values of the 3,544 SNPs are shown in **Figure 1**. The phagocytosis capacity phenotypes (measured through FITC) and the phenotypes for the proportion of phagocytic cells were highly correlated (**Figure 1A** and **Table S2**), and clustered together, jointly with NEU and NLR (**Figure 1B**). Haematological erythrocyte-related measurements had also a high positive correlation, similar to the ones reported for the white blood cell types (**Table S2**). These phenotypes were separated in a second cluster, with HB, HCT and ERY being sub-clustered together and in close proximity to MCH and MCV, and all leukocyte-related counts but NEU (i.e., LEU, EO, MON and LYM) were grouped together in a differentiated branch (**Figure 1B**). Associated to them, the last sub-cluster gathered most of the innate immunity traits such as positive correlated acute-phase proteins (CRP and HP) or positive correlated immunoglobulins concentrations (IgA, IgG and IgM) in plasma, but also other phenotypes as CORT, PLA or MCHC. The latter showed a high correlation with IgA measured in saliva (**Figure 1A** and **Table S2**). The percentage of γδ T cells, the key phenotype used in the AWM approach, was also enclosed in this last sub-cluster, but far from the rest of phenotypes, since it was not correlated with any phenotype (**Figure 1A**).

The gene co-association network was comprised of 3,636 nodes and 451,407 interactions (**Figure S1**). The topology of the network reflected an average degree of 248.299, a clustering coefficient of 0.347 and an average path length of 2.219, which manifest a great level of connection between the nodes of the network. Of note, the three genes with the highest degree (i.e., the highest number of connections) were *REPS2*, *ACSL3* and *WLS*, with 893, 858 and 837, respectively. *ACSL3* and *WLS* knockout mice showed abnormal hematopoietic and immune responses, including decreased γδ T cell number in the latter (37).

### Key Transcription Factors Regulating Health-Related Traits

After PCIT/network analysis, we identified a total of 246 potential regulators, including three microRNAs, within the 3,636 co-associated genes for the 30 health-related traits. The information lossless approach (12) allowed identifying trios of regulators with maximum connectivity (i.e., interactions) within the network; the top 10 trios are shown in **Table 2**. Among them, those composed by *BRMS1L*, *MED12L* and *SUPT3H*, and by *ARNT2*, *SUPT3H* and *TRIM25*, were among the top trios that spanned most of the network topology with highest connectivity (1,482 and 1,472 unique interactions, respectively) and minimum redundancy (**Table 2**). *BRMS1L* and *TRIM25* were the regulators with the highest number of co-associated genes (n=603), followed by *SUPT3H* (n=580), *ARNT2* (n=552), and *MED12L* (n=466). All these protein-coding genes are transcription factors with repressor (*BRMS1L*), co-activator (*MED12L*), or activator functions. For each significant association between a SNP of the five key transcription factors and a trait, the allele substitution effect of the SNP on the respective phenotype is summarised in **Table S3**.

**Table 2:** Top 10 regulator trios based by the number of unique interactions.

Trio	No. of interactions
***BRMS1L - MED12L - SUPT3H***	1482
***MED12L - NCOR2 - SUPT3H***	1476
***ARNT2 - SUPT3H - TRIM25***	1472
***ATF2 - BRMS1L - SUPT3H***	1471
***L3MBTL2 - NCOR2 - SUPT3H***	1470
***NCOR2 - SUPT3H - TRIM25***	1469
***ATF2 - NCOR2 - SUPT3H***	1464
***L3MBTL2 - PHOX2A - SUPT3H***	1463
***ARNT2 - L3MBTL2 - SUPT3H***	1461
***ARNT2 - ESR1 - SUPT3H***	1454

### Functional Classification and Identification of Candidate Genes Associated to Immunocompetence

A functional classification considering all the genes (n=1,828) co-associated with the five top regulators was performed to identify biological processes, metabolic pathways and candidate genes directly related to immunocompetence and study their role in immune system processes. The list of immune biological processes and pathways identified is shown in **Table S4**. Overall, genes for immune cells activation, differentiation and proliferation, regulation of innate and adaptive immune responses, phagocytosis, immunoglobulin and cytokine production and regulation of inflammatory response processes were identified. It is worth to highlight the JAK/STAT pathway, the ERK1/2 and MAPK cascades, and the NOTCH and Phospholipase D signaling pathways among those processes. Furthermore, we also identified other metabolic pathways directly related to immunometabolism such as the AMPK, mTOR, PI3K-Akt and PPAR signaling pathways, which control oxidative phosphorylation, carbohydrate, lipid and peptide metabolic processes, among others. This functional analysis revealed that a total of 589 candidate genes belonged to immune-related functions (**Table S4**).

**Figure 2:**
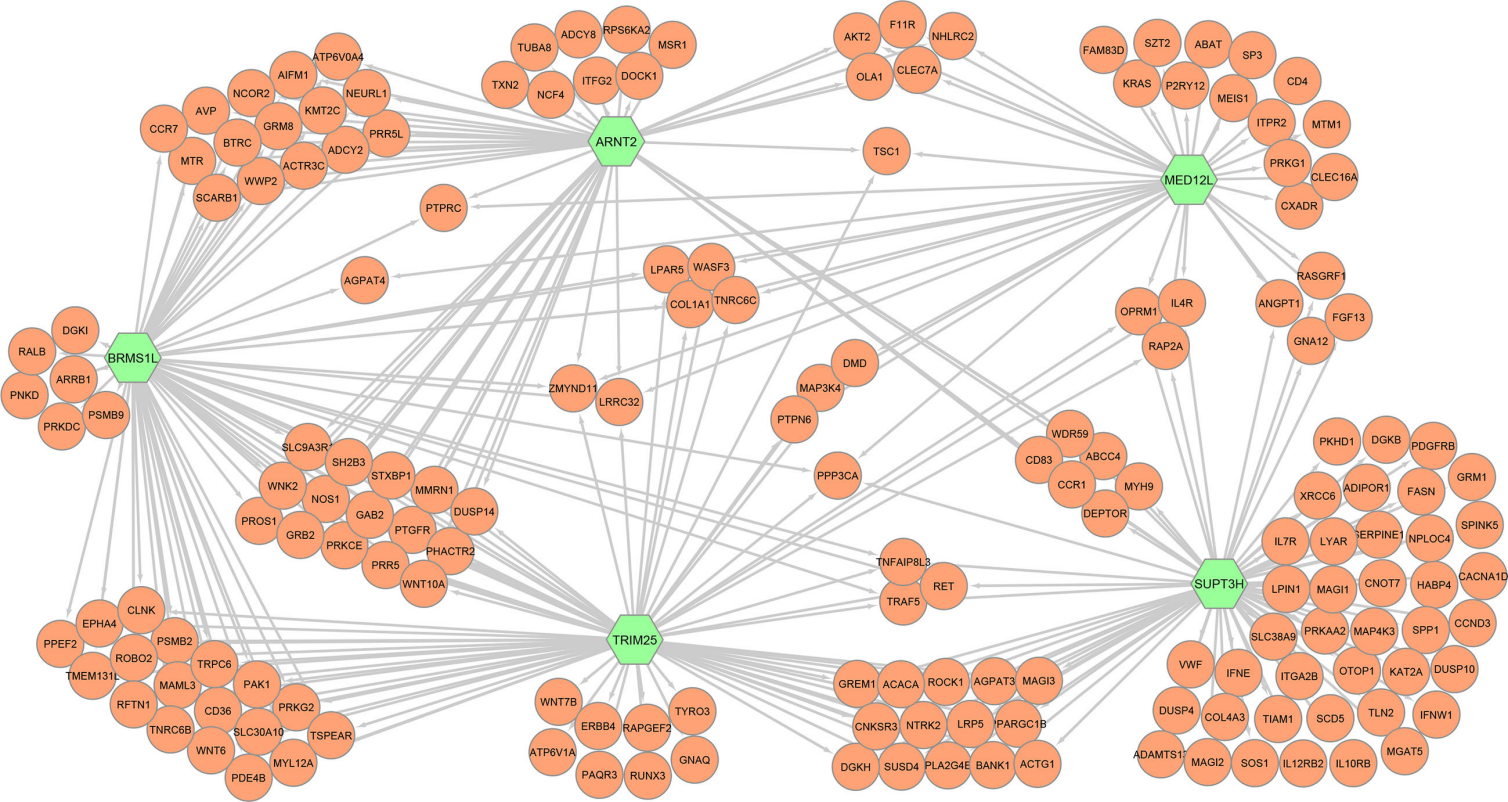
Simplification of the full gene co-association network formed by 171 nodes and 832 interactions representing the immunity-associated genes (orange) that were correlated with the five top regulator genes (green; *ARNT2*, *BRMS1L*, *MED12L*, *SUPT3H*, and *TRIM25*).

In addition, we studied in detail for each top transcription factor those immune processes in which a high number of co-associated genes were involved (**Table S5**). **Figure 2** shows a simplified co-association network representing those immunity-associated genes interacting with the top 5 regulators. **Table 3** summarizes the main immune and metabolic processes in which the top transcription factors were associated. It is worthy to note that all the regulators were co-associated with genes related to platelet functions and phospholipase D signaling. Furthermore, among the top five regulators SUPT3H was the transcription factor co-associated with the highest number of pathways and processes related to immunometabolism and haemopoiesis and immune development, and BRMS1L was the only transcription factor co-associated with genes related to the NOTCH signaling pathway. Finally, we identified a specific gene, protein tyrosine phosphatase receptor type C (*PTPRC*), involved in γδ T cell differentiation and activation that was co-associated with *ARNT2*, *BRMS1L* and *MED12L*.

**Table 3:** Summary of the main immune and metabolic processes associated with the top transcription factors.

Immune and metabolic processes	Top transcription factors
***Immune processes:***	
Platelet-related functions: megakaryocyte differentiation, platelets activation, degranulation, aggregation	*ARNT2, BRMS1L, MED12L, SUPT3H, TRIM25*
Phagocytosis-related functions: phagosome, Fc gamma R-mediated phagocytosis, response to nitric oxide	*ARNT2, BRMS1L, TRIM25*
γδ T cell differentiation and activation	*ARNT2, BRMS1L, MED12L*
Leukocyte differentiation and activation	*SUPT3H, TRIM25*
T and B-cell related functions: T cell differentiation, T and B cell receptor signaling	*BRMS1L, MED12L, SUPT3H*
Positive regulation of haemopoiesis	*SUPT3H*
NOTCH signaling pathway	*BRMS1L*
MAPK signaling pathway	*BRMS1L, SUPT3H, TRIM25*
ERK1 and ERK2 cascade	*SUPT3H, TRIM25*
JAK/STAT pathway	*SUPT3H*
Phospholipase D signaling	*ARNT2, BRMS1L, MED12L, SUPT3H, TRIM25*
***Metabolic processes:***	
mTOR signaling pathway	*ARNT2, MED12L, SUPT3H, TRIM25*
AMPK signaling pathway	*SUPT3H*
PI3K-Akt signaling pathway	*SUPT3H*

### Comparison With Literature Reported Candidate Genes and QTLs for Health-Related Traits

When we studied in detail the top five regulators and their co-associated genes related to immune functions, we identified interesting candidate genes enclosed in genomic regions previously associated with immune phenotypes in studies with humans and pigs (**Table S6**). For instance, *PRKCE*, a candidate gene for haematological parameters in humans (38–41) and different leukocyte subpopulations in pigs (42), was associated in our study with phagocytosis-related traits. Other genes also associated with phagocytosis-related traits in our population were *CD36* and *SH2B3*. In humans, *CD36* has been associated with haematological traits (38, 39), while *SH2B3* has been associated with a plethora of immune phenotypes, mainly with leukocyte levels and their types (38–40). *MYH9* was another candidate gene associated with haematological parameters in humans (38–40) together with *ANGPT1* (38, 39, 43), where the latter was also associated with the levels of exhaled nitric oxide and IgG glycosylation in serum (44, 45). In our analysis, *MYH9* was associated with LYM and phagocytosis-related traits while *ANGPT1* was also associated with NO and some haematological traits. The previously mentioned *PTPRC* (also known as *CD45*) was associated in humans with leukocyte, lymphocyte and monocyte counts (38, 39, 43) and memory T cell differentiation (46). In our population this gene was associated with LYM, among other traits. Other candidate genes commonly associated with immune cell types in humans were: *ATXN1* and *CLEC16A* (38–40, 43), *CD4* (46), *FTO* and *KLRK1* (47); while in pigs were *AKT2* (48), *CLEC16A* (42), *JMJD1C* and *RBFOX1* (8), *PRKG2* and *PAQR3* (49), and *SLC30A10* (50). *CD4* and *KLRK1* are mainly expressed in T cells and Natural Killer cells and they were associated with γδ T cells in our population. Another interesting gene described in association with CRP in humans (51) and with platelet levels in pigs (50) was *PDE4B.* This gene was associated with haematological and phagocytosis-related traits in our study.

Furthermore, while none of the top five regulators were located in QTL regions associated with health-related traits in our Duroc pig population (7), they were located in QTLs for C3c concentration, HCT, HB, Interleukin (IL)-2 and IL-10 levels, Toll-like receptor (TLR)-2 and TLR-9 levels, and basophil number, among others (**Table S6**), in other pig populations (35).

## Discussion

In the present study, a systems genetic approach using the AWM methodology was used to identify potential regulators, gene-gene interactions and pathways for immunocompetence in pigs considering 30 health-related phenotypes enriched for innate immune phenotypes. We selected γδ T cells as the key phenotype, since these cells play important roles at the interface between innate and adaptive immunity and had the potential to impact other phenotypes analysed in our population. In fact, in a previous study in the same population (7), positive and negative genetic correlations were identified between this phenotype and acute phase proteins and platelets (positive correlations), and IgAsal, PHAGO-FITC and cortisol levels (negative correlations). Similar functions such as response to pathogens and tumours, wound healing, tissue homeostasis, stressed cell clearance, and antigen presentation, among others, have been described for γδ T cells in several species [reviewed in Holderness et al. (52)]. However, compared to humans and mice, this population of cells is strongly enriched in pig blood (53), which also suggest specific immune responses and functions.

After GWAS analysis and the SNP annotation step, only 3,544 SNPs were retained for further analysis. When compared with the heritability values obtained with the whole dataset of SNPs, this subset of 3,544 SNPs helped to explain a greater proportion of the additive variance of the analysed traits. As expected, the heritability of our key phenotype (γδ T cells) was greatly improved from moderate to high after using the reduced dataset, despite other phenotypes such as HB and HCT had a greater improvement and a higher heritability value. This may also be due to the fact that they were the two phenotypes with the highest number of associated SNPs after the key phenotype. Among the associated SNPs with HB and HCT, we identified a pleiotropic SNP (*rs81284215*) that was associated with more than 10 phenotypes and was located in the *HBB* gene. It is well established the relationship between HB levels and growth on the early-stages of life (54, 55). Similarly, a positive association between HB and HCT and average daily weight gain has also been described in pigs (56). Therefore, this SNP could be a useful marker to determine the haematological status of our animals, although further analysis will be required to determine the relationship of this polymorphism with weight gain in piglets.

Interestingly, the AWM additive effects correlations of the 3,544 SNPs primarily clustered health-related traits according to their nature and function and were informative enough to capture the genetic associations among the health-related traits in a similar way than the published genetic correlations calculated for the same population (7). Of note, there was a lack of genetic correlation among our key phenotype and the rest of the health-related phenotypes with 225 SNPs exclusively associated with γδ T cells. However, we also identified 534 SNPs associated with γδ T cells and one or more health-related phenotypes, highlighting the genetic interplay between several immune pathways and functions. A possible explanation for these results is the multitasking role of γδ T cells in the immune system with a conventional adaptive role, but with increasing relevance in their influences on innate immunity (57, 58). In fact, it has been proposed a direct but also and indirect role of γδ T cells in promoting monocyte and macrophage differentiation and macrophage recruitment during inflammatory response, as well as in macrophage elimination during the resolution of inflammation (57). Additionally, a specialized role of epithelial γδ T cells in wound repair by promoting platelet and neutrophil accumulation in the limbal vessels has been proposed (59). Overall, these functions are the reflection of the results found during this work that will be discussed below.

In this study, AWM co-association analysis allowed the identification of five key regulators (*ARNT2*, *BRMS1L*, *MED12L*, *SUPT3H* and *TRIM25*) for immunocompetence in pigs. It is relevant to note that for most of these regulators (*BRMS1L*, *MED12L*, *SUPT3H*) there is still little information in the literature about their relationship with immunity-related functions, despite all regulators were located within QTL regions associated to immunity phenotypes (pigQTLdb). Among the identified top regulators, *BRMS1L* and *TRIM25* gathered the highest number of associations with other genes. While *BRMS1L* has been described as a metastatic suppressor gene (60), *TRIM25* has been involved in numerous cellular processes including regulation of innate immune response against viral infection (61, 62). Specifically, *TRIM25* is involved in the RIG-I/interferon pathway in response to viral RNAs (63). Among the list of genes regulated by TRIM25, we identified two genes (*MMP12* and *PTPN6*) related to regulation of type I interferon-mediated signaling pathway. Another key regulator that also plays a role in the innate immune system was ARNT2. This transcription factor forms heterodimers with the aryl hydrocarbon receptor (AHR), that is expressed in all γδ T cell lymphocyte subsets, being essential for some aspects of their functions [reviewed in Stange and Veldhoen (64)]. This complex regulates the transcription of multiple immune-related genes and the activity and differentiation of phagocytic cells and lymphocytes (65). Accordingly, this transcription factor was associated with leukocyte, lymphocyte and monocyte counts in our population and was co-associated with the *PTPRC* gene that was related to γδ T cell differentiation. Furthermore, a polymorphism in *ARNT2* has been associated with an impaired fungicidal activity by depleting the phagocytic activity of macrophages (66). Notably, in our gene-network we identified ARNT2-regulated genes involved in FcγR-mediated phagocytosis (*ACTR3C*, *AKT2*, *DOCK1*, *GAB2*, *PRKCE*, *PTPRC*) and response to nitric oxide (*AIFM1*, *CCR7*, *MTR*, *TXN2*) processes. Therefore, these genes are interesting candidate genes to contribute to the phenotypic differences observed for these health-related traits. Consistently with our results, some of these genes (*AKT2*, *PRKCE*, *PTPRC*) were previously associated with health-related traits in humans and/or pigs, and seven of the ten genes have been at least associated with a phagocytosis-related trait in our population. *MED12L* is a paralog of the *MED12* gene, which is required to activate innate immunity genes in *Drosophila* (67). A recent GWAS study identified a variant within the *MED12L* gene associated with viral infection response (Epstein-Barr virus (EBV) nuclear antigen (EBNA)) in humans (68). In the network, the *MED12L* gene was also interacting with genes associated to γδ T cell differentiation (*PTPRC*) and activation (*CXADR* and *PTPRC*). Finally, a genetic study found an association between a polymorphism in the *SUPT3H* gene with the ADAMTS13 activity, a protein with antithrombotic properties (69).

Except for some genes directly related to the key phenotype (γδ T cell), the five regulators and their 1,828 co-associated genes were involved in a wide variety of biological functions, some of them directly related to immunity but also to immunometabolism. Among them, there were functions related to lymphocytes (differentiation and activation), platelets (activation, degranulation and megakaryocyte differentiation), phagocytosis (FcγR-mediated phagocytosis), NO (response to nitric oxide), and to a lesser extent other functions associated with other immune phenotypes (such as those related to haematological related traits) were identified. Remarkably, for some of these phenotypes (platelets count, NO in serum, and some phagocytosis traits) we did not identify any significantly associated genomic region after performing GWAS (7). Here, it is relevant to highlight that for some of these traits, we identified more than one of the top transcription factors co-associated with genes related to the same functions. Therefore, a plausible explanation for our results would be that due to the complex nature of immunity traits with the involvement of multiple genes and regulators, GWAS analyses are not able to capture all the significant regions associated with their phenotypic variation, since many of the biologically relevant polymorphisms are lost after multiple test corrections. For instance, it is worthy to note the overrepresentation of platelet associated functions, which were commonly found for all the key identified regulators. Apart from being important for coagulation and fibrinolysis processes, platelets are also activators of the immune system and mediators of inflammatory response [reviewed in Trzeciak-Ryczek et al. (70)], therefore participating in multiple functions. In fact, in the hierarchical analysis, platelets phenotype grouped within a heterologous group composed by stress indicators, acute phase proteins and immunoglobulins among others, which may be indicative of its multifunctional nature. Another overrepresented pathway regulated by all the key transcription factors was phospholipase D (PLD) signaling pathway. PLD is an enzyme that catalyses the hydrolysis of phosphatidylcholine, to produce the signal molecule phosphatidic acid (PA). PLD participates in multiple functions of the immune system mediating phagocytosis by macrophages, activation of NADPH oxidation in neutrophils, mast cell degranulation and T cell activation [reviewed in Zhu et al. (71)]. Finally, it is worth mentioning some specific immune functions for the poorly characterized *BRMS1L* and *SUPT3H* regulators; for instance, the regulation of the NOTCH signaling pathway identified for *BRMS1L*. NOTCH signaling plays a pivotal role in T cell fate decision, influencing the differentiation between αβ T cells *versus* γδ T cells (72, 73). Furthermore, *SUPT3H* seems to have an active role in immunometabolism by controlling signaling pathways such as AMPK, mTOR and PI3K-Akt. In recent years, immunometabolism has emerged as an important mechanism affecting innate and adaptive immune system. In fact, AMPK and PI3K/Akt/mTOR regulate metabolic pathways important for cell fate and function in the immune system (74–76). Overall, these results underline the relevance of the five key transcription factors identified in our network regulating important functions of the immune system in pigs. Furthermore, and taking into account the plethora of different functions that these pathways/molecules perform, we could hypothesize, that depending on the function, either one or the interaction of several key transcription factors will be required.

Among the genes co-associated with the top five regulators, we also identified candidate genes that have been previously described as candidate genes for health-related traits in humans and pigs, some of them associated with similar immunological and haematological traits (*ANGPT1, PTPRC, CD4, KLRK1*, and *FTO*). In addition, there were also candidate genes with functions directly related to the associated traits. Indeed, *PRKCE, SH2B3* and *CD36* were associated in our study with phagocytosis-related traits. *PRKCE* plays a role in FcγR-mediated phagocytosis (77), while CD36 is a phagocytic receptor that mediates the uptake of different substrates (78, 79). *SH2B3* is involved in macrophage function (80), whereas *MYH9*, which encodes a conventional non-muscle myosin and is required for normal T lymphocyte migration (81), was associated with the quantity of lymphocytes. *ANGPT1*, a candidate gene in humans, belongs to the angiopoietin family regulating vascular integrity and angiogenesis through endothelium-derived NO release (82), and accordingly, it was also found in association with NO and some haematological traits in our pig population. Also, it is relevant to highlight that the candidate gene *PTPRC* was associated with the quantity of lymphocytes in blood. This member of the protein tyrosine phosphatase (PTP) family is expressed in all nucleated haematopoietic cells and plays an essential role in T and B cell antigen receptor signaling, and it has been associated to many autoimmune disorders [reviewed in Ulloa-Aguirre et al. (83)]. Finally, *PDE4B*, which was associated with haematological and phagocytosis-related traits in our study, is essential for the LPS-activated immune response of phagocytic cells (82, 84).

In conclusion, the AWM gene co-association analysis applied in the present study represented an important step to better characterize the genetic architecture of complex traits for immunity in pigs. The network-based approach allowed the identification of five key transcription factors (*ARNT2*, *BRMS1L*, *MED12L*, *SUPT3H* and *TRIM25*) regulating pathways directly involved in cell fate and in metabolic and immune functions. Furthermore, we have identified gene-gene interactions and candidate genes, some of which have been previously associated to immunity traits in humans and pigs. This study has been the first one to use AWM for analysing health-related traits in pigs and supports the effectiveness of using network-based approaches to identify potential regulators and candidate genes, representing a major step in understanding the regulatory mechanisms involved in porcine immunity, and reinforcing the use of the pig as biomedical model.

## Data Availability Statement

The original contributions presented in the study are included in the article/**Supplementary Material**. Further inquiries can be directed to the corresponding authors.

## Ethics Statement

All experimental procedures with pigs were performed according to the Spanish Policy for Animal Protection RD 53/2013, which meets the European Union Directive 2010/63/EU about the protection of animals used in experimentation. The experimental protocol was approved by the Ethical Committee of the Institut de Recerca i Tecnologia Agroalimentàries (IRTA).

## Author Contributions

RQ and MB designed the study. MB supervised the generation of the animal material used in this work. YR-C, OG-R, MP, RQ and MB performed the sampling. OG-R and MB carried out the laboratory analyses. DC-P, YR-C, RQ and MB analysed the data and interpreted the results. DC-P and MB wrote the manuscript. All authors contributed to the article and approved the submitted version.

## Funding

The study was funded by grants AGL2016-75432-R and PID2020-112677RB-C21 awarded by the Spanish Ministry of Economy and Competitiveness (MINECO). YR-C was funded by Marie Skłodowska-Curie grant (P-Sphere) agreement No 6655919 530 (EU). MB was financially supported by a Ramon y Cajal contract (RYC-2013-12573) from the Spanish Ministry of Economy and Competitiveness. The authors belonged to a Consolidated Research Group AGAUR, ref. 2017SGR-1719.

## Conflict of Interest

The authors declare that the research was conducted in the absence of any commercial or financial relationships that could be construed as a potential conflict of interest.

## Publisher’s Note

All claims expressed in this article are solely those of the authors and do not necessarily represent those of their affiliated organizations, or those of the publisher, the editors and the reviewers. Any product that may be evaluated in this article, or claim that may be made by its manufacturer, is not guaranteed or endorsed by the publisher.

## Glossary

**Table:**

AHR	aryl hydrocarbon receptor
AWM	association weight matrix
CORT	cortisol
CRP	C-reactive protein
EBNA	EBV nuclear antigen
EBV	Epstein-Barr virus
EO	total number of eosinophils
ERY	total number of erythrocytes
FGF12	fibroblast growth factor 12
FITC	fluorescein isothiocyanate
γδ T cells	gamma-delta T cells
GCTA	Genome-wide Complex Trait Analysis
GRANU_PHAGO_%	percentage of phagocytic cells among granulocytes
GRANU_PHAGO_FITC	mean fluorescence in FITC among the granulocytes that phagocyte
GWAS	genome wide association studies
h^2^	heritability value
HB	haemoglobin
HBB	haemoglobin subunit beta
HCT	haematocrit
HP	haptoglobin
Ig	immunoglobulin
IgAsal	IgA in saliva
IL	Interleukin
LEU	total number of leukocytes
LYM	total number of lymphocytes
LYM_PHAGO_%	percentage of phagocytic cells among lymphocytes
LYM_PHAGO_FITC	mean fluorescence in FITC among the lymphocytes that phagocyte
MCH	mean corpuscular haemoglobin
MCHC	mean corpuscular haemoglobin concentration
MCV	mean corpuscular volume
MON	total number of monocytes
MON_PHAGO_%	percentage of phagocytic cells among monocytes
MON_PHAGO_FITC	mean fluorescence in FITC among the monocytes that phagocyte
NEU	total number of neutrophils
NLR	neutrophil to lymphocyte ratio
NO	nitric oxide
PA	phosphatidic acid
PCIT	Partial Correlation and Information Theory
PHAGO_%	percentage of total phagocytic cells
PHAGO_FITC	mean fluorescence in FITC among the total phagocytic cells
PLA	total number of platelets
PLD	phospholipase D
PTP	protein tyrosine phosphatase
PTPRC	phosphatase receptor type C
QTLs	quantitative trait loci
REML	restricted maximum likelihood
SE	standard error
SNPs	single nucleotide polymorphisms
SSC	*Sus scrofa* chromosome
TLR	Toll-like receptor
