# Supplementary material for: A Co-Association Network Analysis Reveals Putative Regulators for Health-Related Traits in Pigs
Source: Front Immunol. 2021 Nov 26;12:784978. doi: 10.3389/fimmu.2021.784978 (PMC8662732; doi:10.3389/fimmu.2021.784978)
Supplement: Supplementary file 1 [file DataSheet_1.docx]

Supplementary Material

# Supplementary Figures and Tables

**Table S1.** Descriptive statistics of the 30 health-related traits analysed in the study (modified from Ballester et al. (2020)).

**Table S2.** Correlation values between the 30 studied phenotypes based on the normalized additive values of the 3,544 SNPs as it is represented in Figure 1B.

**Table S3.** Allele substitution effects (ASE) between the SNPs located on the five key transcription factors and the phenotypes that were associated with them.

**Table S4.** List of the immune biological processes and pathways identified, and the 589 candidate genes found within them, after performing a functional classification considering all the genes (n=1,828) co-associated with the five top regulators.

**Table S5.** List of the immune processes in which a high number of co-associated genes with each one of the five top regulators were involved.

**Table S6.** List of genomic regions associated with immune phenotypes in previous studies with humans and pigs and the candidate genes of our study located within them.

## Supplementary Figures


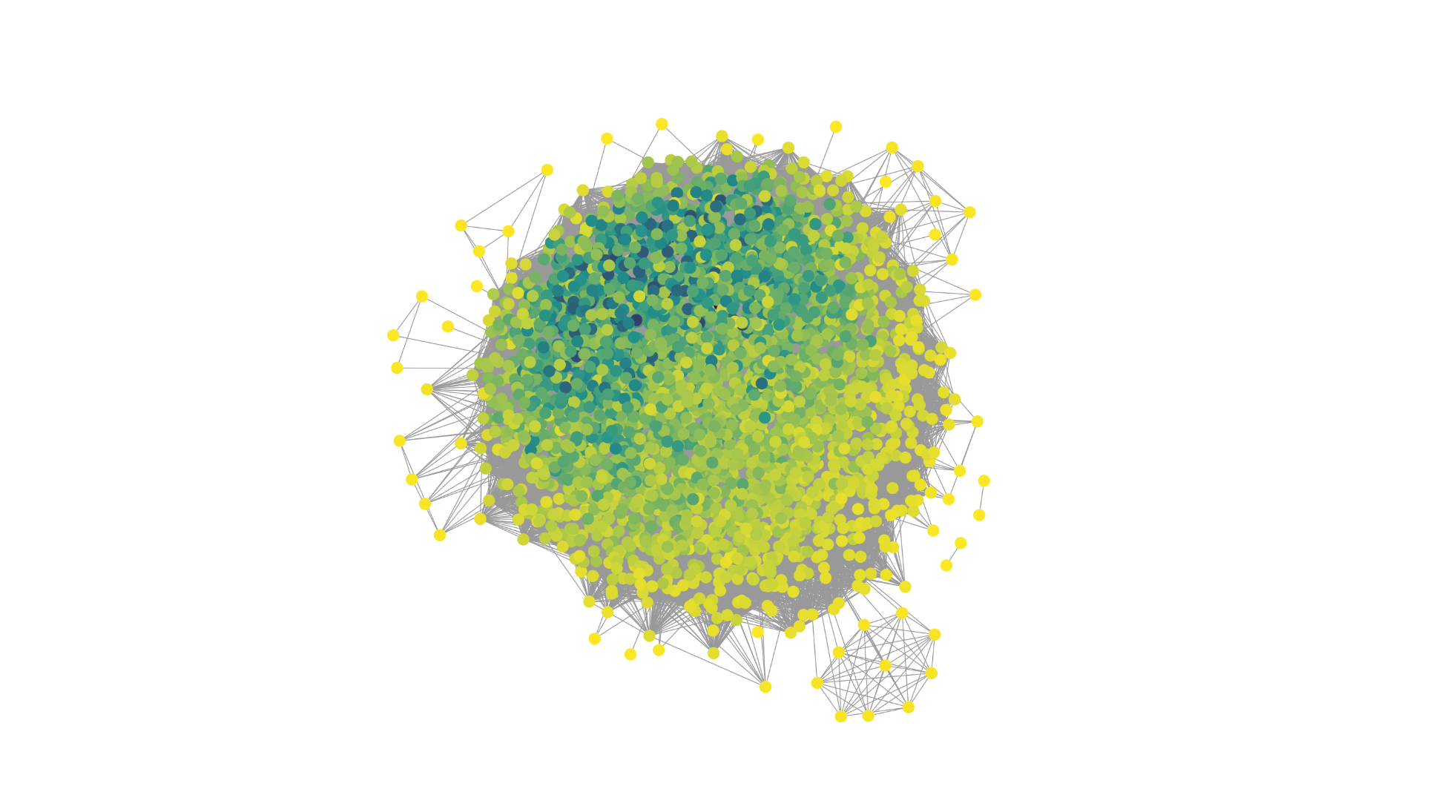


**Figure S1.** Gene co-association network comprised of 3,636 nodes and 451,407 interactions based on the AWM approach. The colour represents the number of interactions for each node from yellow (few interactions) to dark blue (high number of interactions).
